# Supplementary material for: KaMLs for Predicting Protein pKa Values and Ionization States: Are Trees All You Need?
Source: bioRxiv. 2025 Jan 13:2024.11.09.622800. Originally published 2024 Nov 11. Preprint. [Version 2] doi: 10.1101/2024.11.09.622800 (PMC11601431; doi:10.1101/2024.11.09.622800)
Supplement: Supplementary file 1 [file media-1.pdf]

Supporting Information: KaMLs for Predicting Protein pK_a Values and Ionization States: Are Trees All You Need?

Mingzhe Shen,[†] Daniel Kortzak,[†,§] Simon Ambrozak,[‡] Shubham Bhatnagar,[‡] Ian Buchanan,[¶] Ruibin Liu,[†] and Jana Shen[*,†]

† *Department of Pharmaceutical Sciences, University of Maryland School of Pharmacy, Baltimore, MD 21201*

‡ *Department of Computer Science, University of Maryland College Park, College Park, MD 20742*

¶ *Stuyvesant High School, New York, NY 10282*

§ *Joint first author*

E-mail: jana.shen@rx.umaryland.edu

Materials and Methods

PKAD-3 database

Constructing PKAD-3 with unique residue identifications. The current protein pK_a database PKAD-2[S1] contains 1,742 entries associated with 211 proteins. After removing the pK_a's without a specific value, e.g., pK_a >11 or pK_a <2, as well as those of termini, 1,569 entries remain. For these entries, we manually verified the accuracy by reading the source literature. The incorrect pK_a values and residue IDs were corrected. For example, the pK_a of Glu172 in the WT xylanase (associated with PDB entry 1XNB) was corrected from 7.2 to 6.7. The residues that are not resolved in the recorded PDB file (e.g., Glu142 on chain A of the PDB file 1STN) were removed. The cleaned PKAD-2 contains 1,286 entries, which are associated with 763 pK_a's of 615 residues in 113 proteins.

Next, we conducted an exhaustive search for additional experimental pK_a's in peer-reviewed articles published before April 2023. First, we applied keyword pK_a in the title and/or abstract to identify reference articles on the PubMed website. Next, we manually searched for pK_a values in the articles. If the authors of the article associated the pK_a's with an X-ray crystal structure, we recorded its PDB ID. If such information was unavailable, we utilized the UniProt ID of the protein to identify an X-ray structure from the RCSB PDB website.[S2] Additionally, we included 79 pK_a's of mutated Asp, Glu, and Lys of the WT or stabilized constructs (PHS and Δ+PHS) of staphylococcal nuclease (SNase). These pK_a's (documented in Ref.[S3]) were derived from thermodynamic stability measurements conducted by the Garcia-Moreno lab and released in the 2009 blinded protein pK_a prediction challenge.[S4] The uncertainty of these pK_a's was estimated to be 0.2–0.5 units,[S5] similar to the pK_a's determined by NMR shifts.[S6] We designated the cleaned and significantly expanded database as PKAD-3 (Table 1) in maintaining continuity with the original database name PKAD[S7] and PKAD-2.[S1]

To make PKAD-3 ML ready and prevent data leakage (across training, validation, and

test datasets) caused by the different numbering of residues in different PDB files, entries are annotated by the UniProt ID of the protein and PDBrenum[S8] was used to renumber the residue IDs according to the UniProt sequence. Thus, each residue is identified as UniProt ID+Uni_resid. Note that previous ML model developments[S9–S12] have a data leakage issue, in which some residues with different IDs in different PDB files were treated as distinct residues, while in fact they are the same. Below we discuss other steps undertaken to ensure the data is accurate and ML ready.

Mutant proteins and multiple conformational states. Another potential source of data leakage in training ML models is related to annotation of mutant entries. PKAD-3 contains 186 pK_a entries associated with 97 residues in mutants of 6 proteins (Uniprot IDs: P0AEG4, P03069, P00720, P00644, P00282, P09850). Additionally, 100 pK_a entries are associated with 40 residues in 8 proteins (Uniprot IDs: P10599, P68871, P69905, P02945, P00392, Q72F37, P00760, P80028) that have multiple conformational states. These entries were verified to ensure that the X-ray structures are correctly associated with the protein or the conformational state of interest.

To prevent leakage across the training, validation, test datasets, mutant proteins are annotated as 'Uniprot ID-mutation or conformational state', e.g., P09850-Q7E for mutant Q7E; P10599-1 and P10599-2 for two conformational states. A special case is SNase, which has three background constructs (WT, PHS, and Δ+PHS) for engineered mutations. In this case, we use, for example, P00644-V66D, P00644-WT-V66D, and P00644-PHS-V66D to denote the Δ+PHS, WT< and PHS constructs, respectively.

Entries associated with the same residue but different pK_a's. We verified the pK_a entries associated with the same protein residue but differ by more than 1 unit. In certain instances, the large pK_a difference is due to a mutation in the protein, in which case, we verified that the mutant is represented by an appropriate X-ray structure or a computational model (see Modeling of mutant structures). In other instances, the large pK_a

difference is due to a conformational change, in which case we verified that the conformational state discussed in the literature is indeed represented by the recorded PDB entry. For example, in the oxidized state of thioredoxin (PDB entry 1ERU), the pK_a of Asp26 is 8.1, while in the reduced state the pK_a is 9.9 (PDB entry 1ERT).[S13] In some instances, the large pK_a difference cannot be “reconciled”. For example, Glu172 has two measured pK_a's, 7.2 (PDB entry 1XNB) and 2.8 (PDB entry 3VZO). In the latter case, a nearby residue Glu78 is covalently linked to the substrate and another nearby residue is mutated, which likely results in the large pK_a downshift of Glu172. Since ligand binding is not accounted for by the ML models, the pK_a entry 2.8 was removed. For Asp85 in bacteriorhodopsin, the reported pK_a's are 2.6 (PDB: 1UCQ) and 7.2 (PDB: 1IW6). We removed the former entry, since retinol in the 1UCQ structure forms a salt bridge with Asp85. For Asp102 in trypsin, the reported pK_a's are 1.4 (PDB: 2TGA)[S14] and 6.8 (PDB: 1C5Q).[S15] We removed latter pK_a, as it was later assigned to the coupling partner His57.[S14]

Excluded pK_a values. The pK_a's of residues interacting with HEME or ligand (e.g., PDB: 3VZO, Glu172) were excluded, as the ML models do not account for HEME or ligand. Furthermore, we removed Glu325 of LacY (PDB: 2V8N) with a reported pK_a of 10.5.[S16] It is the only Glu with such an extreme (6.3 unit) pK_a shift relative to the model, and the value itself is suspicious. Sugar transport of LacY is coupled to proton co-transport involving Glu325. However, a pK_a of 10.5 makes de-protonation unlikely. Furthermore, the pK_a of Glu325 has been suggested to depend on the conformation of LacY.[S16] In light of these considerations, we decided to exclude Glu325 of LacY in PKAD-3.

Preparation of structures for ML training

Protein structure files were downloaded from the PDB. The PDBfixer function from OpenMM[S17] was used to add missing atoms/residues and remove ligands and waters. The PDBrenum

software[S8] was used to renumber the residue IDs according to the UniProt sequence[S18] to ensure that each residue together with the protein Uniprot ID is assigned a unique identification. For multimer proteins, the monomer that contains the residue with experiment pK_a was retained. Unless X-ray structure is available, the structure of a mutant protein was modeled by SWISS-MODEL[S19] using the X-ray structure of the WT (or the appropriate background construct in the case of SNase) as the template.

Data augmentation using AlphaFold2 models. AlphaFold2 (AF2)[S20] predictions were made using the colabfold_batch program from the ColabFold v1.5.5[S21] docker image. The multiple sequence alignment (MSA) sampling was carried out on the MMSeqs2[S22] server and the results were downloaded for the remainder of the calculations. MSA sampling used 32 cluster centers and 64 extra sequences. We used 32 random seeds and enabled dropout at the default rate for more varied structures. Lastly, we recycled each structure three times, since higher recycling did not increase the template-modeling (pTM) scores. The default parameters were used for all other ColabFold parameters.

Development of KaML-trees and KaML-GATs

Data splitting protocol. The "StratifiedGroupKFold" strategy adapted from sklearn[S23] is applied to randomly sample 10% of PKA3-D as the hold-out test set. Employing the same strategy, the remaining 90% is divided into training and validation sets in a 9:1 ratio. To ensure that the models are evaluated with statistical uncertainties, the data splitting process will be repeated 20 times to generate 20 independent ML models. The same training and test data are used for KaML-trees and KaML-GATs.

A unique group is defined as a combination of UniProt ID+Uni_resid. Stratification was performed on the pK_a's to ensure a similar distribution between the training and test datasets. Specifically, for each amino acid, we manually binned the unique residues based on their pK_a's. The bin width was defined such that residues with different pK_a's

can be separated while most of the bins can be further split into training, validation, and test sets (SI Figure S1).

Feature engineering for the KaML-trees. To describe the local environment of the titratable residue, we adapted six types of features used by the tree models for cysteine ligandability predictions:[S24]

- basic characteristics of the titratable residue;
- solvent accessibility;
- potential hydrogen bond (h-bond) interactions;
- potential salt-bridge interactions;
- propensity of local disorder;
- secondary structure;

A complete list of the features and explanations is given in Table S1.

Training the KaML-trees. For training KaML-trees, the labels were the absolute pK_a's, and the training loss function was the mean squared error (MSE). Model training was repeated 20 times with 20 sets of training/validation and test datasets split as discussed above. 10-fold cross-validation was employed. The open-source Python libraries scikit-learn,[S23] Pycaret,[S25] and CatBoost[S26] were employed for model training and hyperparameter optimization. The latter utilized the random search algorithm with 5000 iterations. The optimized hyperparameters were used to train a final model based on the entire PKAD-3, and the model was saved for user deployment.

AF2 structure augmentation. Out of the 32 AF2 predicted structures for each protein, we only added those in a similar conformation as the X-ray structure. The conformational similarity was defined by the numerical features of the tree models. First, all numerical features were min-max normalized. Next, a similarity score S(f1,f2) was calculated as the

maximum of the squared differences.

$$S(f_1, f_2) = \max_{1 \leq i \leq N_{\times features}} \{(f_{1i} - f_{2i})^2, \} \quad (1)$$

where f1 and f2 refer to the features of the X-ray and AF2 structures, respectively. We tested different augmentation schemes defined by the threshold of pK_a shifts (> 1 or > 2), the similarity cut-off (0.04 or 0.01) as well as a maximum number of AF2 structures to add (at most 10 or all within the similarity cut-off).

Calculation of data irregularity. To investigate feature irregularity, we followed the approach used by McElfresh and coworkers.[S27] The Python package PyMFE[S28] was used to extract meta-features from the tree features of the entire PKAD-3 dataset. From the meta-features, an irregularity score (6.3) was calculated as weighted sum of the smallest eigenvalue of the covariance matrix (weight of -0.33), the skewness of the standard deviation (weight of 0.23), the skewness of the range (weight of 0.22), the interquartile range of the harmonic mean (weight 0f 0.21), and the standard deviation of the kurtosis (weight of 0.21).

Graph representation of the protein structures. The Python package Graphein[S29] was used to build graphs that represent the titratable residues. For computational efficiency, similar to the 20-Å cubic box used in DeepKa,[S30,S31] the protein structure was truncated using BioPandas[S32] as a sphere of 10-Å radius around the most relevant atoms in the titratable sidechain: OD1/OD2 for Asp, OE1/OE2 for Glu, ND1/NE2 for His, SG for Cys, NZ for Lys, and OH for Tyr. Within this sphere, all atoms were considered nodes. The graph incorporates 7 edge types based on pairwise atomic distances. Bonded edges connect atom pairs within 2.2 Å, while non-bonded edges link atoms separated by 2.2-3.5 Å. A bonded edge has 3 subtypes: single, double (based on the distance cutoffs[S33]), and ring (edge within a cycle). A non-bonded edge (with a cutoff of 3.5 Å) has 4 subtypes:

hydrophobic (between Ala, Val, Leu, Ile, Met, Phe, Trp, Pro, and Tyr), aromatic (between Phe, Trp, His, and Tyr), ionic (between Arg, Lys, His, Asp, and Glu), and hydrogen bond (h-bond). A h-bond is searched between sidechain h-bond donors: ARG (NE, NH1, NH2), ASN (ND2), GLN (NE2), HIS (ND1, NE2), LYS (NZ), SER (OG), THR (OG1), TRP (NE1), or TYR (OH) and h-bond acceptors: ASN (OD1), ASP (OD1, OD2), GLN (OE1), GLU (OE1, OE2), or HIS (ND1, NE2). Currently, the backbone atoms and Cys sulfur are not considered in building h-bond edges by Graphein,[S29] which is a limitation and will be addressed in our future work.

We devised a 42-digit-vector node embedding scheme similar to the one used in the CNN model for predicting cysteine ligandabilities.[S24]

- 24-digit one-hot encoding for the atom type of the CHARMM c22 force field[S34] (see SI Table S6);
- 1 integer to indicate if the atom belongs to the titratable residue (0: no; 1: yes);
- 7-digit one-hot for amino acid: Asp, Glu, Cys, Tyr, His, Lys, or other;
- 1 integer for the atom hybridization state;
- 1 integer for the number of bonded heavy atoms;
- 1 integer for the number of hetero atoms bonded;
- 1 float for the atom partial charge from the CHARMM c22 force field;[S34]
- 1 float for the model pK_a (Asp 3.7, Glu 4.2, His 6.5, Cys 8.5, Tyr 10.1, and Lys 10.4);
- 5-digit one-hot to indicate hydrophobic, aromatic, h-bond acceptor, h-bond donor, or ring.

Architecture and hyperparameters of the KaML-GATs. The PyTorch Geometric (PyG) library[S35] was used to build the KaML-GATs (Figure 5 in the main text). The KaML-GAT contains 3 one-head convolutional (conv) layers, each having 42 channels based on the graph attention operator.[S36] The attention coefficient between the ith and the jth node can

be expressed as

$$\alpha_{ij} = \frac{\exp(LeakyReLU(\overrightarrow{a}^T[W\overrightarrow{h}_i||W\overrightarrow{h}_j]))}{\sum_{k\in N_i}\exp(LeakyReLU(\overrightarrow{a}^T[W\overrightarrow{h}_i||W\overrightarrow{h}_k]))}, \quad (2)$$

where $\overrightarrow{a}^T$ is the attention coefficient, W is the linear transformation weight, and || denotes the aggregation function. The LeakyReLU is an activation function defined as:

$$LeakyRelu(x) = max(0, x) + 0.2 * min(0, x). \quad (3)$$

A hyperbolic tangent (tanh) layer is added after each conv layer to add non-linearity to the model. A global mean pooling layer is applied to the output of the last conv layer to represent the whole graph embedding. The pooling results are flattened to a vector of length 42 and fed to the fully connected (FC) layer. The FC layer is made up of 2 hidden layers containing 32 and 16 neurons. The rectified linear unit (ReLU) activation function functions is applied to all the hidden layers. Finally, 1 FC layer containing 1 neuron with a linear activation function is used to make prediction. Batch normalization is applied to all the layers except the last FC layer. A training batch size of 64 and a learning rate of 0.0005 was used. A dropout rate of 0.2 was applied to both conv and FC layers. The Adam optimizer was used for minimizing training loss MSE. To prevent over-fitting, early stopping was applied if the validation loss stops decreasing in the next 50 epochs with a tolerance of 0.1. The total number of parameters in KaML-GAT is ~ 8000.

Training the KaML-GATs. The Pytorch API[S37] was used to implement model training. Training labels were the experimental (in training) or calculated (in pre-training) pK_a shifts relative to the model values, while in analysis, the shifts were converted back to the pK_a's. Model pre-training utilized PHMD549,[S31] a dataset comprised of Asp, Glu, His, and Lys pK_a's calculated by the pH-replica exchange[S38] GBNeck2-CpHMD[S39] simulations. After removing proteins in the PKAD-3 database, there were 26,252 pK_a's of 25,912 residues

in 535 proteins. Note, this dataset does not contain pK_a's of Cys and Tyr, and the highly shifted pK_a's may not be accurate due to the short simulation time.[S31] During pre-training, the model weights were initialized using the Glorot algorithm.[S40]

Once pre-training was completed, different layer freezing schemes were tested (see main text) before training on the experimental pK_a shifts. For each data split, 10 GATs were trained using different 9:1 train-CV splits independent of each other. The models with the validation RMSE $<$ 1.2 were selected to form an ensemble GAT for hold-out testing.

A web portal for the PKAD-3 database

We implemented a web portal (https://database.computchem.org/pkad-3). Each entry contains the PDB ID, chain ID, residue type, residue ID (in the PDB file), pK_a value, Uniprot_id, Uniprot_resid. The Note column contains information about mutation and/or protein conformational state. Hyperlinks for the cited references are also included. Users can search the PKAD-3 database by specific columns (e.g., PDB, Chain) and apply criterion to filter pK_a values that meet certain criteria (e.g., > 7). Users can also select specific rows from their search results. The user selection, the entire search result, or the complete database can be downloaded as a csv file.

Supplemental Tables

Table S1: Features used for the decision tree ML models

Feature name	Description
	**Characteristics of the titratable residue**
res_type	residue type
model_pK_a	pK_a of model compound in solution
charge	residue charge at physiological pH
n_sc_C	number of side chain carbon atom
	**Solvent Accessibility**
buried_ratio	1 - SASA(X)/SASA(model)
d0/1_polar	distance from the sidechain COM to the first/second nearest polar atom
d0/1_nonpolar	distance from the sidechain COM to the first/second nearest nonpolar atom
n_polar_5/10/15	number of polar atoms within 5/10/15Å of the side chain COM
n_nonpolar_5/10/15	number of nonpolar atoms within 5/10/15Å of the sidechain COM
n_hv_6/9/12/15	number of heavy atoms within 6/9/12/15Å of the sidechain COM
	**Potential h-bond interactions**
d0/1_hbond_O	distance from the sidechain COM to the first/second nearest Ser/Thr/Tyr side chain O
d0/1_hbond_N	distance from the side chain COM to the first/second nearest Asn/Gln/Trp sidechain N
d0/1_hbond_H	distance from the side chain COM to the first/second nearest His sidechain N
d0/1_C_SG	distance from the sidechain COM to the first and second nearest Cys side chain SG
d0/1_bb_NH	distance from the sidechain COM to the first/second nearest backbone N
	**Potential electrostatic interactions**
d0/1_neg_O	distance from the side chain COM to the first/second nearest Asp/Glu side chain O
d0/1_pos_N	distance from the side chain COM to the first/second nearest Arg/Lys side chain N
	**Protein flexibility**
flexibility	predicted by RIDAO[S41] on a 30-amino acid stretch centered at the titratable residue.
	**Protein secondary structure**
rss	secondary structure of the ROI
rssp2/4	secondary structure of the residue at +2/+4 position
rssm2/4	secondary structure of the residue at -2/-4 position
	**Potential backbone h-bond interactions**
DA1/2	Distance from the backbone N of the titratable residue to the nearest/second nearest backbone O of any other residue
DD1/2	Distance from the backbone O of the titratable residue to the nearest/second nearest backbone N of any other residue

[a] SASA (solvent accessible surface area) is calculated by DSSP.[S42] Model refers to the penta-peptide AAXAA, where X = ROI. A command line version of RIDAO[S41] was used.

Table S2: Overall performance comparison of the different KaML-trees for predicting p$K_a$values

	CB			ET			GB	RF	XGB
	all	acid	base	all	acid	base	all	all	all
PCC	0.94 ± 0.02	0.88 ± 0.03	0.93 ± 0.03	0.94 ± 0.02	0.86 ± 0.03	0.93 ± 0.03	0.94 ± 0.02	0.94 ± 0.02	0.93 ± 0.02
RMSE	0.77 ± 0.09	0.77 ± 0.11	0.76 ± 0.09	0.80 ± 0.10	0.82 ± 0.13	0.74 ± 0.09	0.81 ± 0.07	0.81 ± 0.08	0.86 ± 0.09
MAXE	3.47 ± 0.39	3.25 ± 0.53	2.60 ± 0.70	3.70 ± 0.78	3.61 ± 1.01	2.46 ± 0.74	3.79 ± 0.49	3.66 ± 0.58	4.08 ± 0.85

[a]The metrics of the CBtree are shown in bold font. All models were trained on the non-augmented dataset.

Table S3: Overall performance comparison of the different KaML-trees for predicting protonation states at pH 7[a]

	CB			ET			GB	RF	XGB
	all	acid	base	all	acid	base	all	all	all
Rec^{prot}	**0.93**	0.82	0.96	0.90	0.88	0.91	0.93	0.93	0.93
Pre^{prot}	**0.97**	0.91	0.99	0.95	0.88	0.98	0.96	0.96	0.91
Rec^{dep}	**0.98**	0.99	0.99	0.94	0.99	0.97	0.99	0.99	0.98
Pre^{dep}	**0.99**	0.99	0.95	0.98	0.99	0.88	0.98	0.98	0.98
CER	46/2635	34/2099	12/536	64/2638	30/2061	34/577	46/2578	48/2533	72/2606

[a]The metrics of the CBtree are shown in bold font. All models were trained on the non-augmented dataset.

Table S4: Performance metrics of the acid/base KaML-CBtree with AF2 structure augmentation

	w/o aug	2.0,0.04,all	2.0,0.04,10	2.0,0.01,all	2.0,0.01,10	1.0,0.04,all	1.0,0.04,10	1.0,0.01,all	1.0,0.01,10
PCC	0.94 ± 0.02	0.94 ± 0.02	0.94 ± 0.02	0.94 ± 0.02	0.94 ± 0.02	0.93 ± 0.02	0.94 ± 0.02	0.94 ± 0.02	0.94 ± 0.02
RMSE	0.77 ± 0.09	0.84 ± 0.10	0.81 ± 0.09	0.82 ± 0.08	0.83 ± 0.10	0.85 ± 0.12	0.840.11	0.81 ± 0.08	0.83 ± 0.10
MAXE	4.1 ± 0.85	3.60 ± 0.55	3.8 ± 0.38	3.61 ± 0.42	3.8 ± 0.5	3.7 ± 0.5	3.8 ± 0.4	3.8 ± 0.2	3.7 ± 0.4
Pre^{prot}	0.91	0.90	0.90	0.91	0.92	0.88	0.88	0.91	0.91
Pre^{dep}	0.94	0.94	0.94	0.93	0.93	0.94	0.94	0.95	0.94
Rec^{prot}	0.74	0.75	0.76	0.74	0.74	0.77	0.78	0.75	0.75
Rec^{dep}	0.94	0.93	0.93	0.93	0.93	0.93	0.93	0.94	0.94
CER	46/2635	51/2635	46/2635	41/2635	55/2635	59/2635	57/2635	48/2635	53/2635

Models were trained with augmented dataset, column labels indicate augmentation parameters (ΔpK_a, similarity, number of structures).

Table S5: Performance metrics of KaML-CBtree for individual residue types

	Asp	Glu	His	Cys	Lys	Tyr
PCC	0.86 ± 0.06	0.84 ± 0.04	0.51 ± 0.19	0.61 ± 0.52	0.80 ± 0.16	0.39 ± 0.85
RMSE	0.75 ± 0.17	0.60 ± 0.07	0.85 ± 0.14	1.50 ± 0.60	0.70 ± 0.21	1.24 ± 0.85
Protonation state prediction at pH 7.0						
Prec^{prot}	0.95	1.0	1.0	0.68	0.99	–
Rec^{prot}	0.77	0.81	0.37	0.65	1.0	–
Prec^{dep}	0.99	0.99	0.95	0.73	1.0	–
Rec^{dep}	0.99	1.0	1.0	0.76	0.80	–
CER	13/916	5/1076	11/209	13/68	1/325	
Protonation state prediction at pH 7.5						
Prec^{prot}	0.91	1.0	1.0	0.75	0.99	–
Rec^{prot}	0.67	0.56	0.18	0.38	1.0	–
Prec^{dep}	0.99	0.99	0.98	0.71	1.0	–
Rec^{dep}	0.99	1.0	1.0	0.93	0.80	–
CER	6/896	7/1068	9/390	12/43	2/315	
Number of protonated/deprotonated states in the test set data^a						
N^{prot} (pH 7.0)	62	40	91	31	315	34
N^{dep} (pH 7.0)	888	1032	282	36	10	3
N^{prot} (pH 7.5)	24	22	25	20	313	34
N^{dep} (pH 7.5)	892	1054	417	40	12	3

^{a}The number of protonated/deprotonated states at pH 7 or 7.5 calculated from the true (experimental) pK_a values. Protonation state is defined by the protonation probability: protonated if Prob > 0.75, i.e., p$K_a < 6.52$ at pH 7 or p$K_a < 7$ at pH 7.5; deprotonated if Prob < 0.25, i.e., p$K_a > 7.02$ at pH 7 or p$K_a > 7.98$ at pH 7.5.

Table S6: Performance metrics of PROPKA for individual residue types

	Asp	Glu	His	Cys	Lys	Tyr
PCC	0.64 ± 0.16	0.69 ± 0.12	0.45 ± 0.23	0.12 ± 0.75	0.75 ± 0.16	–
RMSE	1.12 ± 0.16	1.02 ± 0.23	1.03 ± 0.25	3.58 ± 0.81	0.80 ± 0.22	1.67 ± 1.80
Classification at pH 7.0[a]						
Pre^{prot}	0.84	0.67	0.6	0.47	0.98	–
Rec^{prot}	0.28	0.93	0.12	1.0	0.99	–
Pre^{dep}	0.97	0.99	0.85	NaN	0.0	–
Rec^{dep}	0.99	0.98	0.98	0.0	0.0	–
CER	31/907	21/1045	47/303	35/66	6/315	
Classification at pH 7.5[b]						
Pre^{prot}	0.56	0.66	0.0	0.44	0.98	–
Rec^{prot}	0.28	1.0	0.0	1.0	0.99	–
Pre^{dep}	0.99	1.0	1.0	NaN	0.50	–
Rec^{dep}	0.99	0.99	1.0	0.0	0.29	–
CER	17/908	11/1041	29/434	36/64	7/315	-

[a]Classification of protonation states uses the protonation probability given a predicted pK_a, 0–0.25 (or p$K_a > 7.5$) for deprotonated and 0.75–1 (or p$K_a < 6.5$) for protonated. Residues with experimental pK_a outside of this ranges were ignored. [b] Classification of protonation states uses the protonation probability given a predicted pK_a, 0–0.25 (or p$K_a > 8$) for deprotonated and 0.75–1 (or pK_a<7) for protonated, Critical error rate (CER) refers to the percentage cases of miss-classifying 'protonated' as 'deprotonated' or vice versa.

Table S7: CHARMM22 atom types

Atom type	Description
C	Carbonyl C, peptide backbone
CA	Aromatic C
CT1	Aliphatic sp3 C for CH
CT2	Aliphatic sp3 C for CH_2
CT3	Aliphatic sp3 C for CH_3
CPH1	His CG and CD2 carbons
CPH2	His CE1 carbon
CPT	Trp C between rings
CY	Trp C in pyrrole ring
CP1	Tetrahedral C (Pro CA)
CP2	Tetrahedral C (Pro CB/CG)
CP3	Tetrahedral C (Pro CD)
CC	Carbonyl C, Asn, Asp, Gln, Glu, cter, ct2
N	Pro N
NR3	Charged his ring N
NH1	Peptide N
NH2	Amide N
NH3	Ammonium N
NC2	Guanidinium N
NY	Trp N in pyrrole ring
O	Carbonyl O
OC	Carboxylate O
OH1	Hydroxyl O
S	Sulphur

Table S8: Performance metrics of the pre-trained ensemble KaML-GAT model with different layer freezing schemes[a]

	freeze0	freeze1	freeze2
PCC	0.93 ± 0.02	0.92 ± 0.03	0.92 ± 0.04
RMSE	0.90 ± 0.08	0.92 ± 0.09	0.96 ± 0.12
MAXE	3.74 ± 0.49	3.74 ± 0.54	3.90 ± 0.76
Rec^{prot}	0.92 ± 0.06	0.93 ± 0.06	0.89 ± 0.07
Pre^{prot}	0.92 ± 0.05	0.91 ± 0.07	0.90 ± 0.07
Rec^{dep}	0.98 ± 0.01	0.98 ± 0.01	0.98 ± 0.01
Pre^{dep}	0.98 ± 0.01	0.98 ± 0.01	0.98 ± 0.02
CER	$70/2822$	$75/2822$	$94/2822$

[a]Freeze0, 1, 2 indicates freezing none, the first one, and the first two GAT layers after pretraining.

Table S9: Effect of data augmentation and pretraining on the performance of the GAT model

Method	RMSE	PCC
PT + DA	0.84	0.95
DA only	0.95	0.94
PT only	0.93	0.93
No PT/DA	0.90	0.94

Model comparison is based on one training/test split.

Table S10: Performance metrics of the ensemble KaML-GAT for different residue types

	Asp	Glu	His	Cys	Lys	Tyr
PCC	0.82 ± 0.08	0.73 ± 0.07	0.45 ± 0.18	0.59 ± 0.56	0.68 ± 0.21	–
RMSE	0.88 ± 0.17	0.76 ± 0.08	0.86 ± 0.18	1.81 ± 0.56	0.87 ± 0.28	1.79 ± 0.95
Pre^{prot}	0.95	1.00	0.25	0.58	0.97	–
Rec^{prot}	0.75	0.52	0.13	0.97	1.00	–
Pre^{dep}	1.00	0.99	0.92	0.85	0.00	–
Rec^{dep}	1.00	1.00	0.94	0.47	0.00	–

Table S11: Overall performance of the PB and ML models

Method	RMSE
PypKa[S43]	0.82^a
PKAI[S12]	1.15^a
PKAI+[S12]	0.98^a
DeepKa[S30,S31]	1.05^{a,b}
ANI-2X[S10]	n/a^c
KaML-CBtree	0.77±0.07
KaML-GAT	0.90±0.08

^{a} Metrics of alternative models are taken from the references. ^{b} CYS and TYR are not included in the RMSE. ^{c} The overall RMSE is not given in Ref.[S10]

Table S12: Classification metrics of PypKa, DeepKa, and ANI-2X for individual residue types

	Asp	Glu	His	Cys	Lys	Tyr
			PypKa			
MAXE	6.24 ± 1.3	2.81 ± 0.74	3.14 ± 1.49	4.34 ± 1.68	2.78 ± 0.72	1.92 ± 1.59
Pre^{prot}	NaN	1.0	0.94	0.50	0.96	–
Rec^{prot}	0.00	0.24	0.72	1.00	1.00	–
Pre^{dep}	0.94	0.98	0.97	1.00	NaN	–
Rec^{dep}	1.00	1.00	0.99	0.16	0.00	–
			ANI-2X			
MAXE	4.44 ± 0.23	4.05 ± 0.47	1.85 ± 0.66	–	3.86 ± 0.55	2.52 ± 1.71
Pre^{prot}	1.00	Nan	1.00	–	0.96	–
Rec^{prot}	0.09	0.0	0.93	–	1.00	–
Pre^{dep}	0.94	0.96	0.98	–	NaN	–
Rec^{dep}	1.00	1.00	1.00	–	0.00	–
			DeepKa			
Pre^{prot}	0.86	1.00	0.75	–	1.00	–
Rec^{prot}	0.12	0.79	0.65	–	1.00	–
Pre^{dep}	0.95	0.99	0.96	–	0.86	–
Rec^{dep}	1.00	1.00	0.98	–	0.86	–

Supplemental Figures

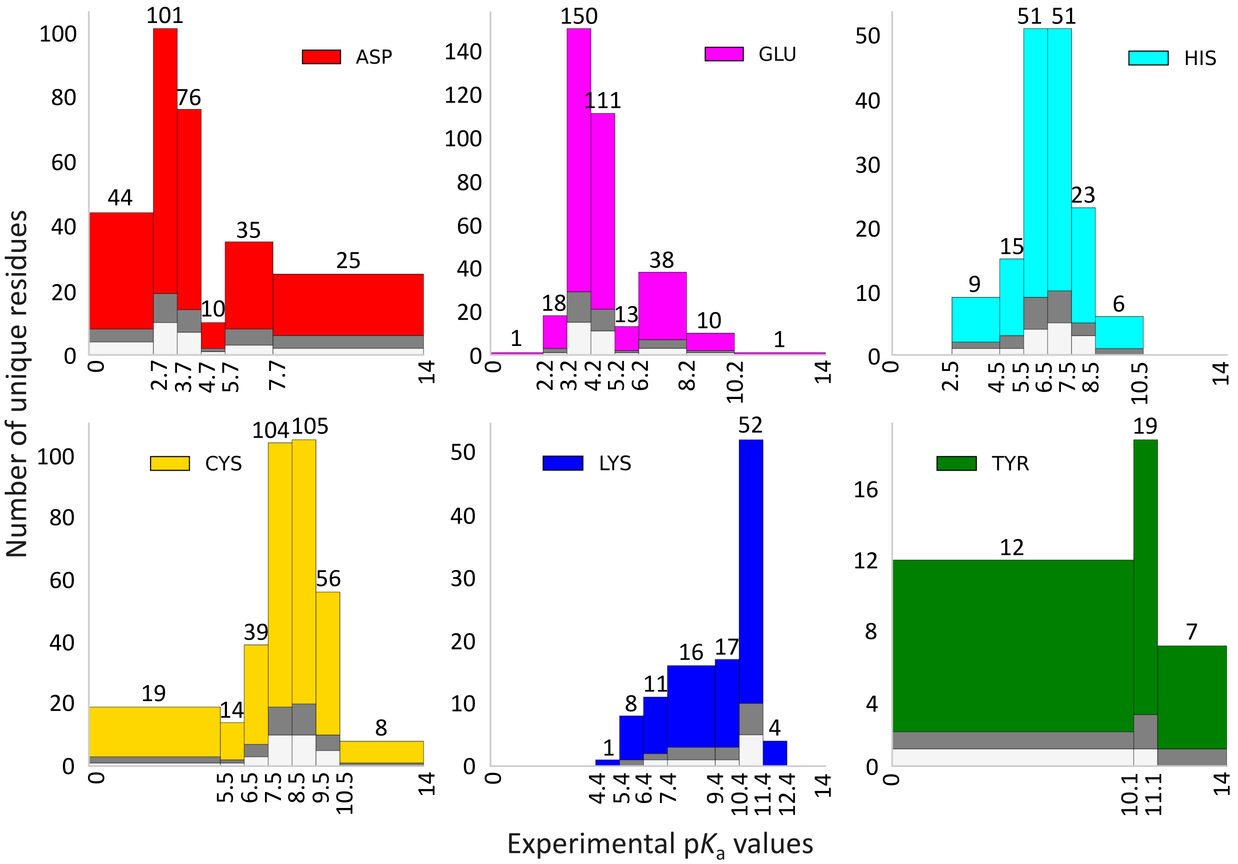

Figure S1: **Train-validation-test split.** The binning scheme used in this study for each residue type. The colored bins represent the overall dataset, grey bins represent the validation dataset, and the white bins represent the test dataset.

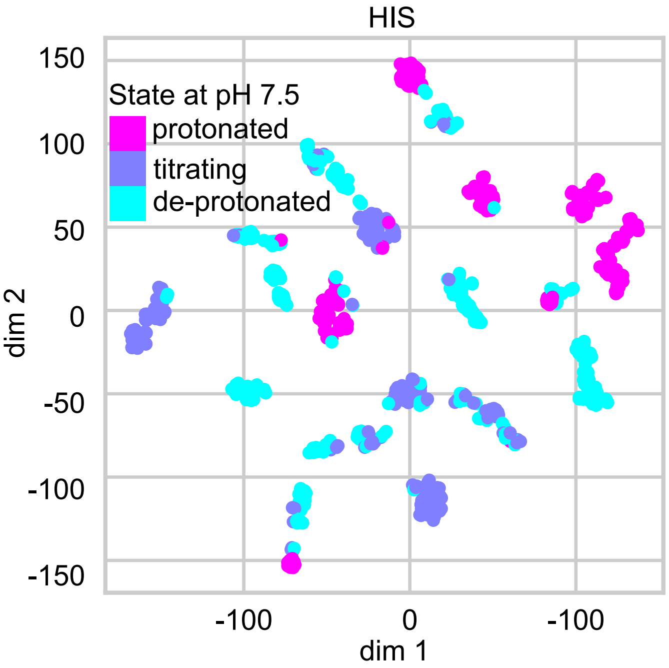

Figure S2: **t-distributed Stochastic Neighbor Embedding (t-SNE) visualization of the histidine data.** The numerical tree features of all histidines were extracted from the dataset with the AF2 augmentation. This 37 dimensional data was then mapped on two dimension using t-SNE. The color code indicates the protonation state at pH 7.0 based on the label experimental pK_a: protonated (p$K_a > 7.5$); deprotonated (p$K_a < 6.5$); titrating ($6.5 \leq$ p$K_a \leq 7.5$).

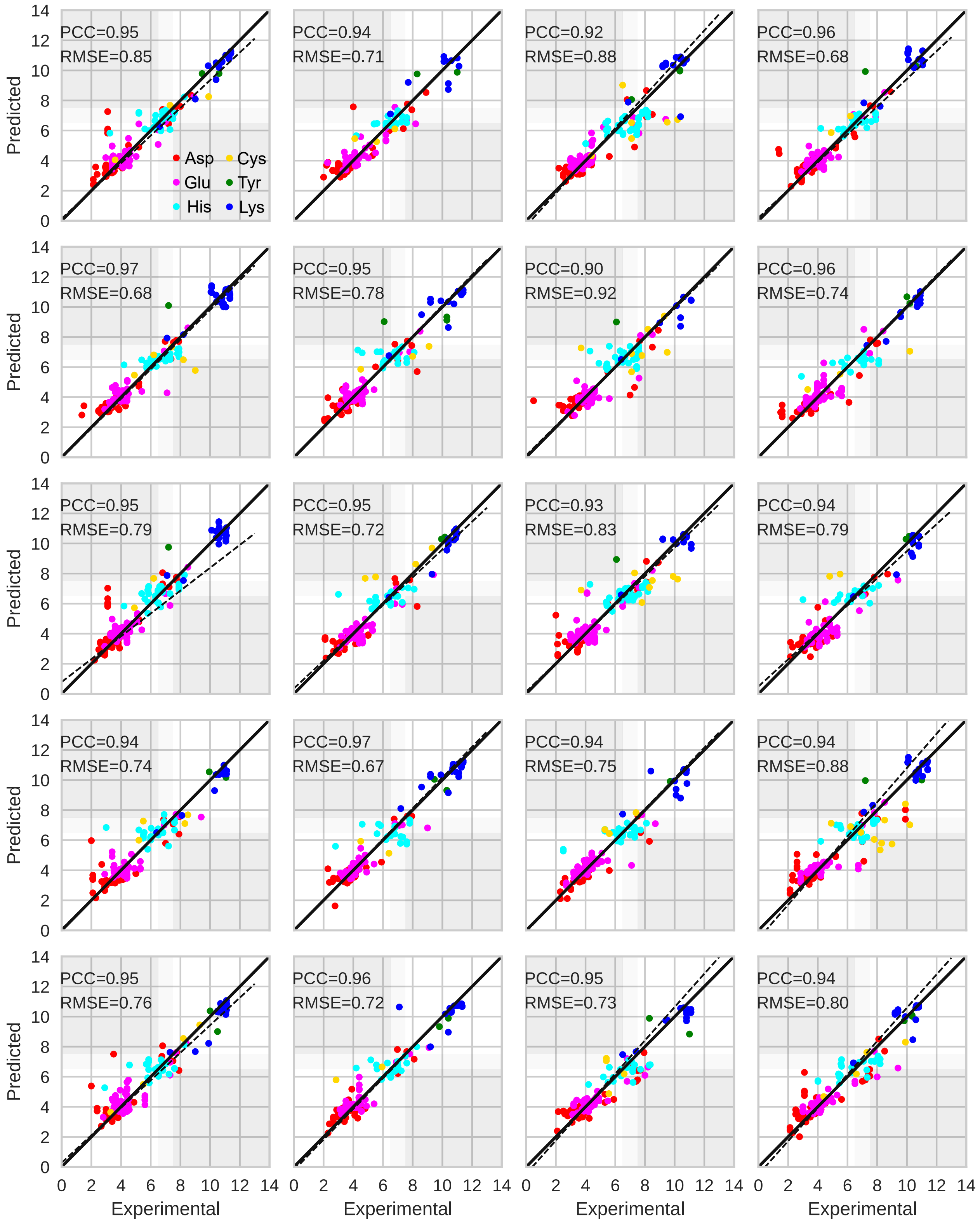

Figure S3: **Comparison of KaML-CBtree predicted and experimental pK_a's in all 20 hold-out tests.** Predicted vs. experimental pK_a values for all test splits. The solid line is the identity, the dashed line is a linear fit.

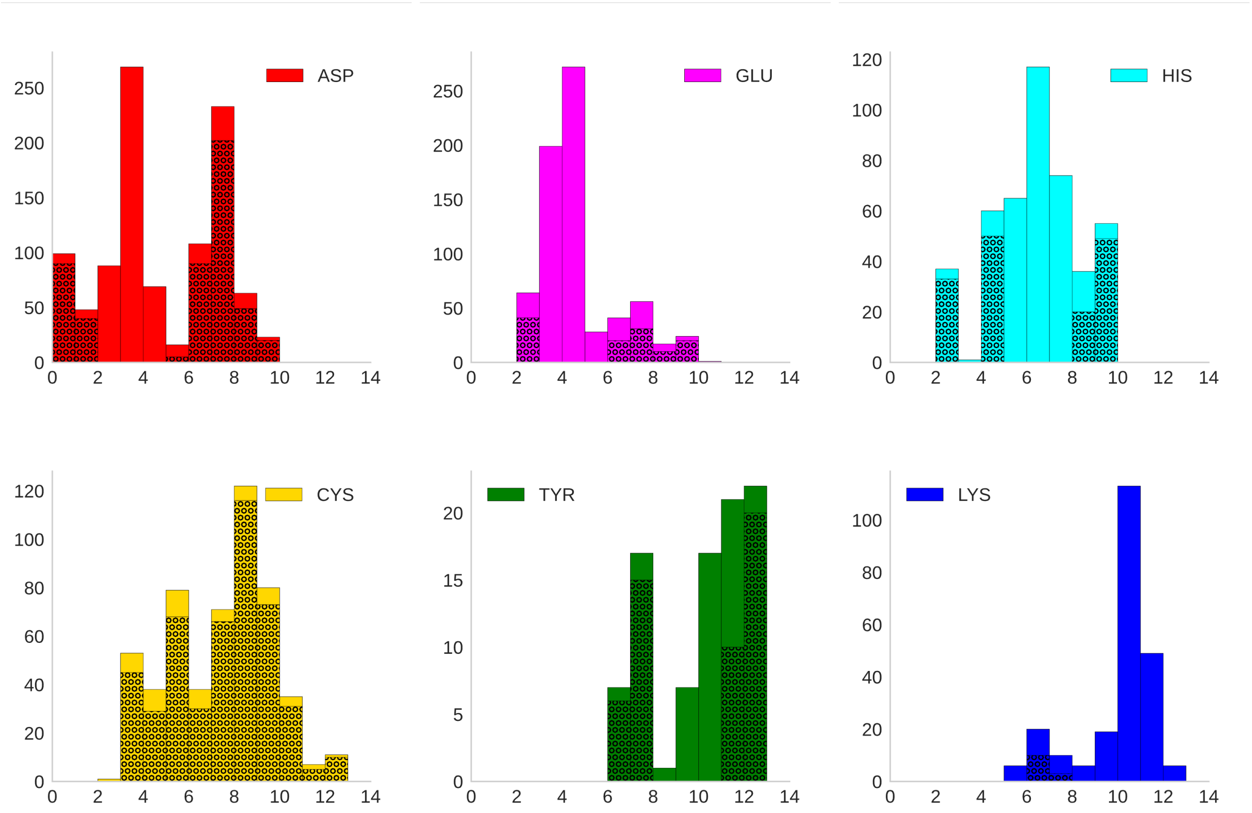

Figure S4: **Histograms of the pK_a values of the training dataset after the AF2 augmentation.** Solid bars represent the total number of entries in the training dataset and shaded bars represent the augmentation using the AF2 structures.

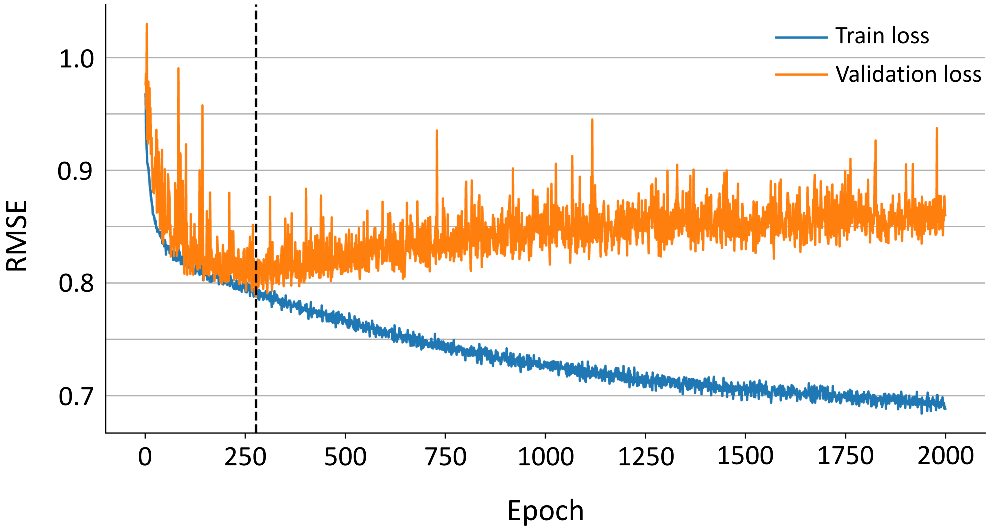

Figure S5: **Training and validation loss in the PHMD549 pre-training.** The dashed vertical line indicates the epoch where the model is saved. The training and validation RMSE is 0.79 and 0.79.

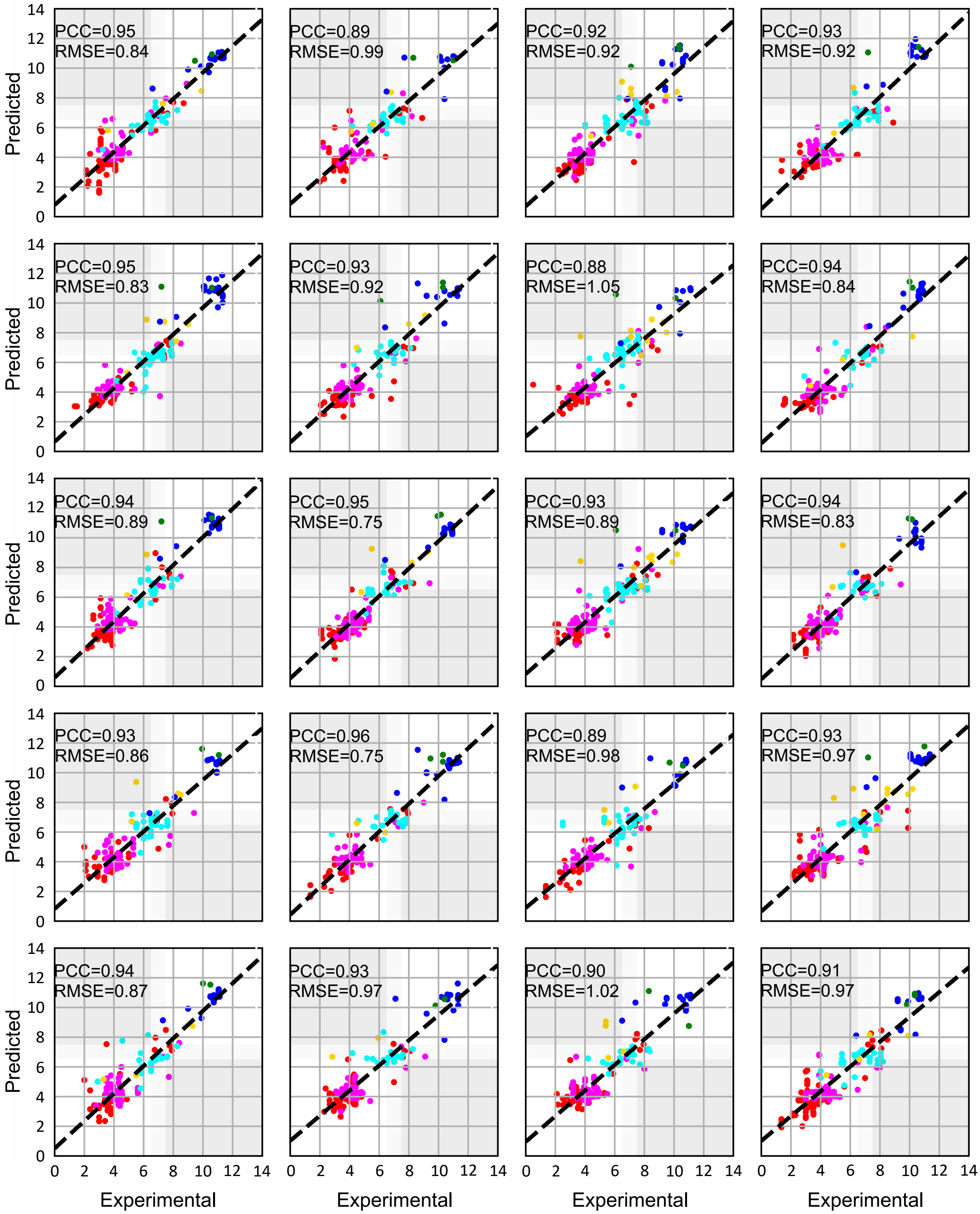

Figure S6: **Comparison of KaML-GAT predicted and experimental pK_a's in all 20 hold-out tests.** Predicted vs. experimental pK_a values for all test splits. The solid line is the identity, the dashed line is a linear fit.

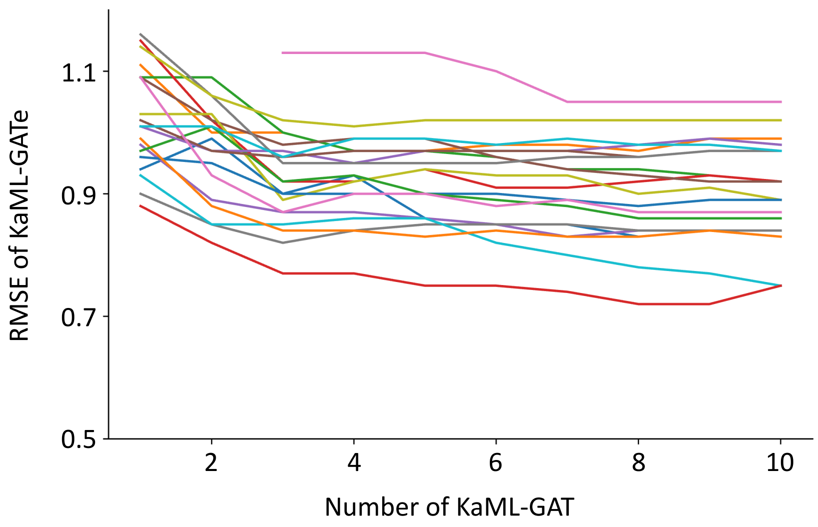

Figure S7: **The influence of the number of KaML-GAT models on the prediction performance of the ensemble KaML-GAT.** The x-axis is the number of KaML-GAT models in the ensemble KaML-GAT. The y-axis is the RMSEs of the ensemble KaML-GAT. The results for the 20 independent tests are shown in different colors.

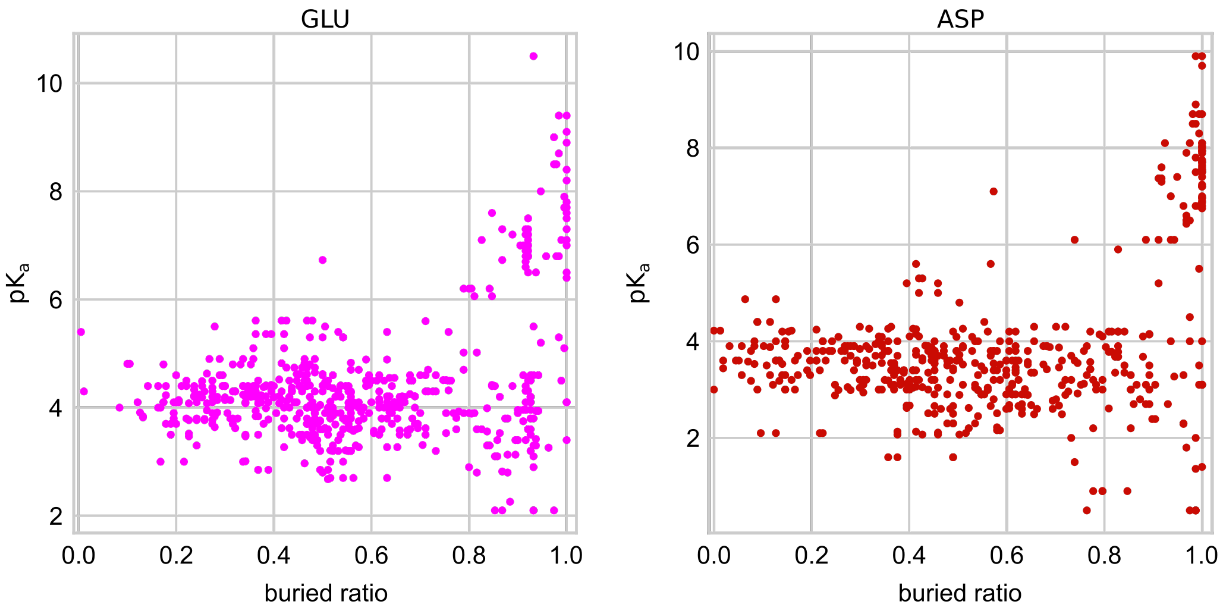

Figure S8: **Glu/Asp with large pK_a values are deeply buried, but some deeply buried Asp/Glu show normal pK_a's.** The experimentally determined pK_a values vs. the calculated buried ratio.

References

(S1) Ancona, N.; Bastola, A.; Alexov, E. PKAD-2: New Entries and Expansion of Functionalities of the Database of Experimentally Measured pK_a's of Proteins. *J. Comput. Biophys. Chem.* **2023**, 1–10.

(S2) Berman, H. M.; Westbrook, J.; Feng, Z.; Gilliland, G.; Bhat, T. N.; Weissig, H.; Shindyalov, I. N.; Bourne, P. E. The Protein Data Bank. *Nucleic Acids Res.* **2000**, *28*, 235–242.

(S3) Wallace, J. A. Modeling Protonation Equilibria at Atomic Resolution: Development and Application of Continuous Constant pH Molecular Dynamics. **2012**,

(S4) Alexov, E.; Mehler, E. L.; Baker, N.; M. Baptista, A.; Huang, Y.; Milletti, F.; Erik Nielsen, J.; Farrell, D.; Carstensen, T.; Olsson, M. H. M.; Shen, J. K.; Warwicker, J.; Williams, S.; Word, J. M. Progress in the Prediction of pK_a Values in Proteins. *Proteins* **2011**, *79*, 3260–3275.

(S5) Isom, D. G.; Castañeda, C. A.; Cannon, B. R.; Velu, P. D.; García-Moreno E., B. Charges in the Hydrophobic Interior of Proteins. *Proc. Natl. Acad. Sci. U.S.A.* **2010**, *107*, 16096–16100.

(S6) Webb, H.; Tynan-Connolly, B. M.; Lee, G. M.; Farrell, D.; O'Meara, F.; Søndergaard, C. R.; Teilum, K.; Hewage, C.; McIntosh, L. P.; Nielsen, J. E. Remeasuring HEWL pKa Values by NMR Spectroscopy: Methods, Analysis, Accuracy, and Implications for Theoretical pKa Calculations. *Proteins* **2011**, *79*, 685–702.

(S7) Pahari, S.; Sun, L.; Basu, S.; Alexov, E. DelPhiPKa: Including Salt in the Calculations and Enabling Polar Residues to Titrate. *Proteins* **2018**, *86*, 1277–1283.

(S8) Faezov, B.; Dunbrack, R. L. PDBrenum: A Webserver and Program Providing Pro-

tein Data Bank Files Renumbered According to Their UniProt Sequences. *PLoS ONE* **2021**, *16*, e0253411.

(S9) Chen, A. Y.; Lee, J.; Damjanovic, A.; Brooks, B. R. Protein pK_a Prediction by Tree-Based Machine Learning. *J. Chem. Theory Comput.* **2022**, *18*, 2673–2686.

(S10) Gokcan, H.; Isayev, O. Prediction of Protein pK_a with Representation Learning. *Chem. Sci.* **2022**, *13*, 2462–2474.

(S11) Liu, S.; Yang, Q.; Zhang, L.; Luo, S. Accurate Protein pK_a Prediction with Physical Organic Chemistry Guided 3D Protein Representation. *J. Chem. Inf. Model.* **2024**, *64*, 4410–4418.

(S12) Reis, P. B.; Bertolini, M.; Montanari, F.; Rocchia, W.; Machuqueiro, M.; Clevert, D.-A. A Fast and Interpretable Deep Learning Approach for Accurate Electrostatics-Driven pK_a Predictions in Proteins. *J. Chem. Theory Comput.* **2022**, *18*, 5068–5078.

(S13) Qin, J.; Clore, G. M.; Gronenborn, A. M. Ionization Equilibria for Side-Chain Carboxyl Groups in Oxidized and Reduced Human Thioredoxin and in the Complex with Its Target Peptide from the Transcription Factor NFκB. *Biochemistry* **1996**, *35*, 7–13.

(S14) Markley, J. L.; Ibanez, I. B. Zymogen Activation in Serine Proteinases. Proton Magnetic Resonance pH Titration Studies of the Two Histidines of Bovine Chymotrypsinogen A and Chymotrypsin A.Alpha. *Biochemistry* **1978**, *17*, 4627–4640.

(S15) Koeppe, R. E.; Stroud, R. M. Mechanism of Hydrolysis by Serine Proteases: Direct Determination of the pK_a's of Aspartyl-102 and Aspartyl-194 in Bovine Trypsin Using Difference Infrared Spectroscopy. *Biochemistry* **1976**, *15*, 3450–3458.

(S16) Grytsyk, N.; Sugihara, J.; Kaback, H. R.; Hellwig, P. pK_a of Glu325 in LacY. *Proc. Natl. Acad. Sci. U.S.A.* **2017**, *114*, 1530–1535.

(S17) Eastman, P.; Swails, J.; Chodera, J. D.; McGibbon, R. T.; Zhao, Y.; Beauchamp, K. A.; Wang, L.-P.; Simmonett, A. C.; Harrigan, M. P.; Stern, C. D.; Wiewiora, R. P.; Brooks, B. R.; Pande, V. S. OpenMM 7: Rapid Development of High Performance Algorithms for Molecular Dynamics. *PLoS Comput. Biol.* **2017**, *13*, e1005659.

(S18) The UniProt Consortium; Bateman, A.; Martin, M.-J.; Orchard, S.; Magrane, M.; Ahmad, S.; Alpi, E.; Bowler-Barnett, E. H.; Britto, R.; Bye-A-Jee, H.; Cukura, A.; Denny, P.; Dogan, T.; Ebenezer, T.; Fan, J.; Garmiri, P.; Da Costa Gonzales, L. J.; Hatton-Ellis, E.; Hussein, A.; Ignatchenko, A.; Insana, G.; Ishtiaq, R.; Joshi, V.; Jyothi, D.; Kandasaamy, S.; Lock, A.; Luciani, A.; Lugaric, M.; Luo, J.; Lussi, Y.; MacDougall, A.; Madeira, F.; Mahmoudy, M.; Mishra, A.; Moulang, K.; Nightingale, A.; Pundir, S.; Qi, G.; Raj, S.; Raposo, P.; Rice, D. L.; Saidi, R.; Santos, R.; Speretta, E.; Stephenson, J.; Totoo, P.; Turner, E.; Tyagi, N.; Vasudev, P.; Warner, K.; Watkins, X.; Zaru, R.; Zellner, H.; Bridge, A. J.; Aimo, L.; Argoud-Puy, G.; Auchincloss, A. H.; Axelsen, K. B.; Bansal, P.; Baratin, D.; Batista Neto, T. M.; Blatter, M.-C.; Bolleman, J. T.; Boutet, E.; Breuza, L.; Gil, B. C.; Casals-Casas, C.; Echioukh, K. C.; Coudert, E.; Cuche, B.; De Castro, E.; Estreicher, A.; Famiglietti, M. L.; Feuermann, M.; Gasteiger, E.; Gaudet, P.; Gehant, S.; Gerritsen, V.; Gos, A.; Gruaz, N.; Hulo, C.; Hyka-Nouspikel, N.; Jungo, F.; Kerhornou, A.; Le Mercier, P.; Lieberherr, D.; Masson, P.; Morgat, A.; Muthukrishnan, V.; Paesano, S.; Pedruzzi, I.; Pilbout, S.; Pourcel, L.; Poux, S.; Pozzato, M.; Pruess, M.; Redaschi, N.; Rivoire, C.; Sigrist, C. J. A.; Sonesson, K. et al. UniProt: The Universal Protein Knowledgebase in 2023. *Nucleic Acids Res.* **2023**, *51*, D523–D531.

(S19) Waterhouse, A.; Bertoni, M.; Bienert, S.; Studer, G.; Tauriello, G.; Gumienny, R.; Heer, F. T.; de Beer, T. A. P.; Rempfer, C.; Bordoli, L.; Lepore, R.; Schwede, T. SWISS-MODEL: Homology Modelling of Protein Structures and Complexes. *Nucleic Acids Res.* **2018**, *46*, W296–W303.

(S20) Jumper, J.; Evans, R.; Pritzel, A.; Green, T.; Figurnov, M.; Ronneberger, O.; Tunyasuvunakool, K.; Bates, R.; Žídek, A.; Potapenko, A.; Bridgland, A.; Meyer, C.; Kohl, S. A. A.; Ballard, A. J.; Cowie, A.; Romera-Paredes, B.; Nikolov, S.; Jain, R.; Adler, J.; Back, T.; Petersen, S.; Reiman, D.; Clancy, E.; Zielinski, M.; Steinegger, M.; Pacholska, M.; Berghammer, T.; Bodenstein, S.; Silver, D.; Vinyals, O.; Senior, A. W.; Kavukcuoglu, K.; Kohli, P.; Hassabis, D. Highly Accurate Protein Structure Prediction with AlphaFold. *Nature* **2021**, *596*, 583–589.

(S21) Mirdita, M.; Schütze, K.; Moriwaki, Y.; Heo, L.; Ovchinnikov, S.; Steinegger, M. ColabFold: Making Protein Folding Accessible to All. *Nat. Methods* **2022**, *19*, 679–682.

(S22) Steinegger, M.; Söding, J. MMseqs2 Enables Sensitive Protein Sequence Searching for the Analysis of Massive Data Sets. *Nat. Biotechnol.* **2017**, *35*, 1026–1028.

(S23) Pedregosa, F.; Varoquaux, G.; Gramfort, A.; Michel, V.; Thirion, B.; Grisel, O.; Blondel, M.; Prettenhofer, P.; Weiss, R.; Dubourg, V.; Vanderplas, J.; Passos, A.; Cournapeau, D.; Brucher, M.; Perrot, M.; Duchesnay, É. Scikit-Learn: Machine Learning in {P}ython. *J. Machine Learn. Res.* **2011**, *12*, 2825–2830.

(S24) Liu, R.; Clayton, J.; Shen, M.; Bhatnagar, S.; Shen, J. Machine Learning Models to Interrogate Proteome-Wide Covalent Ligandabilities Directed at Cysteines. *JACS Au* **2024**, *4*, 1374–1384.

(S25) Ali, M. PyCaret: An Open Source, Low-Code Machine Learning Library in Python. 2020.

(S26) Prokhorenkova, L.; Gusev, G.; Vorobev, A.; Dorogush, A. V.; Gulin, A. CatBoost: unbiased boosting with categorical features. 2019; https://arxiv.org/abs/1706.09516.

(S27) McElfresh, D.; Khandagale, S.; Valverde, J.; Feuer, B.; Hegde, C.; Ramakrishnan, G.; Goldblum, M.; White, C. When Do Neural Nets Outperform Boosted Trees on Tabular Data? 37th Conference on Neural Information Processing Systems. 2023.

(S28) Alcobaça, E.; Siqueira, F.; Rivolli, A.; Garcia, L. P. F.; Oliva, J. T.; de Carvalho, A. C. P. L. F. MFE: Towards reproducible meta-feature extraction. *Journal of Machine Learning Research* **2020**, *21*, 1–5.

(S29) Jamasb, A.; Viñas Torné, R.; Ma, E.; Du, Y.; Harris, C.; Huang, K.; Hall, D.; Lió, P.; Blundell, T. Graphein - a Python Library for Geometric Deep Learning and Network Analysis on Biomolecular Structures and Interaction Networks. Adv. Neural Inf. Process. Syst. 2022; pp 27153–27167.

(S30) Cai, Z.; Luo, F.; Wang, Y.; Li, E.; Huang, Y. Protein pK_a Prediction with Machine Learning. *ACS Omega* **2021**, *6*, 34823–34831.

(S31) Cai, Z.; Liu, T.; Lin, Q.; He, J.; Lei, X.; Luo, F.; Huang, Y. Basis for Accurate Protein pK_a Prediction with Machine Learning. *J. Chem. Inf. Model.* **2023**, *63*, 2936–2947.

(S32) Raschka, S. BioPandas: Working with Molecular Structures in Pandas DataFrames. *The Journal of Open Source Software* **2017**, *2*, 279.

(S33) Baber, J. C.; Hodgkin, E. E. Automatic Assignment of Chemical Connectivity to Organic Molecules in the Cambridge Structural Database. *J. Chem. Inf. Comput. Sci.* **1992**, *32*, 401–406.

(S34) MacKerell, A. D.; Bashford, D.; Bellott, M.; Dunbrack, R. L.; Evanseck, J. D.; Field, M. J.; Fischer, S.; Gao, J.; Guo, H.; Ha, S.; Joseph-McCarthy, D.; Kuchnir, L.; Kuczera, K.; Lau, F. T. K.; Mattos, C.; Michnick, S.; Ngo, T.; Nguyen, D. T.; Prodhom, B.; Reiher, W. E.; Roux, B.; Schlenkrich, M.; Smith, J. C.; Stote, R.; Straub, J.; Watanabe, M.; Wiórkiewicz-Kuczera, J.; Yin, D.; Karplus, M. All-Atom Empirical Potential for Molecular Modeling and Dynamics Studies of Proteins. *J. Phys. Chem. B* **1998**, *102*, 3586–3616.

(S35) Fey, M.; Lenssen, J. E. Fast Graph Representation Learning with PyTorch Geometric. International Conference on Learning Representations. 2019.

(S36) Veličković, P.; Cucurull, G.; Casanova, A.; Romero, A.; Liò, P.; Bengio, Y. Graph Attention Networks. International Conference on Learning Representations. 2018.

(S37) Polikar, R. Ensemble Based Systems in Decision Making. *IEEE Circuits Syst. Mag.* **2006**, *6*, 21–45.

(S38) Wallace, J. A.; Shen, J. K. Continuous Constant pH Molecular Dynamics in Explicit Solvent with pH-Based Replica Exchange. *J. Chem. Theory Comput.* **2011**, *7*, 2617–2629.

(S39) Harris, R. C.; Shen, J. GPU-Accelerated Implementation of Continuous Constant pH Molecular Dynamics in Amber: pK_a Predictions with Single-pH Simulations. *J. Chem. Inf. Model.* **2019**, *59*, 4821–4832.

(S40) Glorot, X.; Bengio, Y. Understanding the Difficulty of Training Deep Feedforward Neural Networks. Proceed. Mach. Learn. Res. 2010.

(S41) Dayhoff II, G. W.; Uversky, V. N. Rapid Prediction and Analysis of Protein Intrinsic Disorder. *Protein Sci.* **2022**, *31*, e4496.

(S42) Kabsch, W.; Sander, C. Dictionary of Protein Secondary Structure: Pattern Recognition of Hydrogen-Bonded and Geomterical Features. *Biopolymers* **1983**, *22*, 2577–2637.

(S43) Reis, P. B. P. S.; Vila-Viçosa, D.; Rocchia, W.; Machuqueiro, M. PypKa: A Flexible Python Module for Poisson–Boltzmann-Based pKa Calculations. *J. Chem. Inf. Model.* **2020**, *60*, 4442–4448.
